# Norwich Lent Assizes, April 1846

**Published:** 1846-07

**Authors:** 


Norwich Lent Assizes, April 1846.

*Trial for the attempt to Procure Abortion. Is White Hellebore a Poison?*—Sarah Whisker was charged with administering to Frances Baily, on the 5th of March last, a small quantity of White Hellebore, for the purpose of procuring abortion.

Mr. Bircham appeared for the prosecution, and having stated the facts to the jury, called

Frances Baily, who stated that she went to the prisoner's house to have her fortune told, and whilst there, the prisoner taxed her with being in the family way, which she admitted, and said, she was seven months gone. The prisoner told her to come in the evening, and she would give her something that would do her good, and not prevent her from going on with her work. She accordingly went, and the prisoner gave her a liquid and a powder, with instructions how to take them. They were taken accordingly, and the result was, that she became very ill and sick. She left her place, and went to Yarmouth, and in a day or two she became so unwell that she was obliged to have medical advice.

Mr. Burgess, surgeon, of Yarmouth, attended the prosecutrix, and stated the situation in which he found her. She was suffering great prostration of body. The stomach was very hot,—the pulse exceedingly low, and the patient evincing a disposition to retch considerably; and on his first seeing her he considered she was in a dangerous state, which induced him, after ascertaining the cause which had produced her illness, to apply to the Mayor upon the subject, with the view to have her deposition taken, and the prisoner apprehended.

Mr. Worship, of Yarmouth, surgeon, was examined as to the condition of the prosecutrix, and Mr. Cook, druggist, of Norwich, who deposed to having frequently sold hellebore to the prisoner. The female servant of the prisoner was called, who deposed to having frequently seen young persons at her mistress's house, and that she had frequently been sent by the prisoner to Mr. Cook's, to purchase white hellebore, hicra picra, dragon's blood, and turpentine.

At the close of the case for the prosecution, Mr. Prendergast took an objection to the indictment, that the evidence did not sustain the count for administering poison, the testimony of the medical gentlemen not establishing that hellebore was a virulent poison. The learned Judge, after much consideration, said, he would consult with Mr. Justice Maule upon it; but the indictment evidently ought to have contained a count for administering poison or some other *noxious* thing.

On the return of his Lordship into court, he expressed a wish to put further questions to Mr. Worship; and on that gentleman being re-called, he further stated, that, in his opinion, hellebore was noxious to the system, and produced ill effects, but he knew of no case in which it had produced death; under these circumstances, he was not justified, he thought, *in calling it a poison.*

Mr. Page Scott, surgeon of the Castle, who happened to come into court at this time, was called upon by the Judge, and asked some questions by his lordship; and he stated, that he was acquainted with hellebore, and, in his opinion, it was a noxious poison. As a medical man, it was decidedly his opinion that *it belonged to the class of drugs called poisons.*

His Lordship then intimated that he should leave the case to the jury.

Mr. Prendergast then addressed the jury for the prisoner; and relied strongly upon the evidence not establishing hellebore as a poison.

His Lordship, in summing up, was of opinion, that *that was a poisonous drug which in common parlance was generally understood and taken to be such;* and the evidence for the prosecution, he thought, was sufficiently strong to bring hellebore within the meaning of the statute.

The jury returned a verdict, "that the prisoner was guilty of administering hellebore with intent to procure abortion."

The Judge—Do you find hellebore to be a poison?—Foreman. The medical men seem to differ on this point; and, therefore, we have not considered that question.—The Judge. You ought to take the way in which, in common understanding, it would be considered You had better consider whether it is a poison or not.

The jury almost immediately found, that it was a poison, but unknown to the prisoner.

His Lordship then had Mr. Cook the druggist, re-called; who stated, that the prisoner had been in the habit of purchasing hellebore at his shop for the last three years. She had purchased from half an ounce to an ounce at a time, and had done so once or twice a week for a considerable time.

A witness was called, who gave the prisoner a good character for being a kind-hearted woman.

His Lordship then proceeded to pass sentence:—The respectable gentleman, observed the learned Judge, who had last given evidence, was not aware he thought, of the practices of the prisoner. She had been found guilty of a very serious offence, and if in any one of the numerous instances of a similar kind in which she had acted, death had been occasioned, she would most undoubtedly have been tried for wilful murder. As the case then stood, he must make an example of her, and pass a severe sentence—as severe as the law would enable him to do; which was, that she be transported beyond the seas for the term of her natural life, as a just reward for one in the catalogue of those numerous offences which too many young women, he feared, if brought forward, would be able to relate.

Remarks.—The most curious feature in this case is the discrepancy in the medical evidence upon a point, concerning which we should have thought no doubt could have existed in the mind of any professional man. Is white hellebore (*Veratrum Album*) a poison, or is it not? One witness admitted that it was noxious to the system, and produced ill effects; but as he had known of no case in which it had produced death, he did not think he was justified in calling it a poison! Fortunately for the ends of justice (the words "noxious thing" having apparently been carelessly omitted in drawing up the indictment) another surgeon happened at this time to come into court, who unhesitatingly gave it as his opinion that it belonged to the class of drugs called poisons.

White hellebore owes its activity to veratria, one of the most formidable among the poisonous alkaloids. The existence of such an alkaloid in white hellebore, if known to the witness, should have satisfied him that it was a poison. If he did not know that it contained veratria, then he was very unfortunately situated to have so material a question put to him. In a very large number of cases, white hellebore powder has been known to act as a powerful irritant, causing violent vomiting and purging, with great prostration of strength. In most of the instances of poisoning by it hitherto observed in the human subject, the persons have recovered; but in one case quoted by Wibmer, a man took as much of the powdered root as would twice cover the point of a knife, vomited seven or eight times, and died the same evening. The same case is quoted by Dr. Christison in the last edition of his work. The medical witness might therefore have readily satisfied himself, by referring to an easily accessible work, that he was decidedly mistaken in his opinion, that in short, white hellebore was not merely *noxious*, but a powerful *poison*. If it produces abortion, it is only by its strong irritant action on the bowels. In the fatal case above quoted from Wibmer, the œsophagus, stomach, and colon, were inflamed. As an additional illustration of its poisonous action, this author relates that twenty grains of the powdered root caused in a dog severe vomiting, purging and convulsions, followed by death in a quarter of an hour. These facts bear out the view taken by Mr. Page Scott. The prisoner, however, nearly escaped conviction; for even the jury were misled by the difference in the medical opinions, on a question on which no reasonable medical doubt can properly exist. The case furnishes in this respect a warning to medical witnesses.—*Med. Gaz.*

